# Lizard movement tracks: variation in path re-use behaviour is consistent with a scent-marking function

**DOI:** 10.7717/peerj.1844

**Published:** 2016-03-22

**Authors:** Stephan T. Leu, Grant Jackson, John F. Roddick, C. Michael Bull

**Affiliations:** 1 School of Biological Sciences, Flinders University, Adelaide, South Australia, Australia; 2 School of Computer Science, Engineering and Mathematics, Flinders University, Adelaide, South Australia, Australia

**Keywords:** Movement strategy, Path re-use, Signalling, Olfactory cues, Lizard, Scincidae, Movement trails

## Abstract

Individual movement influences the spatial and social structuring of a population. Animals regularly use the same paths to move efficiently to familiar places, or to patrol and mark home ranges. We found that Australian sleepy lizards (*Tiliqua rugosa*), a monogamous species with stable pair-bonds, repeatedly used the same paths within their home ranges and investigated whether path re-use functions as a scent-marking behaviour, or whether it is influenced by site familiarity. Lizards can leave scent trails on the substrate when moving through the environment and have a well-developed vomeronasal system to detect and respond to those scents. Path re-use would allow sleepy lizards to concentrate scent marks along these well-used trails, advertising their presence. Hypotheses of mate attraction and mating competition predict that sleepy lizard males, which experience greater intra-sexual competition, mark more strongly. Consistent with those hypotheses, males re-used their paths more than females, and lizards that showed pairing behaviour with individuals of the opposite sex re-used paths more than unpaired lizards, particularly among females. Hinterland marking is most economic when home ranges are large and mobility is low, as is the case in the sleepy lizard. Consistent with this strategy, re-used paths were predominantly located in the inner 50% home range areas. Together, our detailed movement analyses suggest that path re-use is a scent marking behaviour in the sleepy lizard. We also investigated but found less support for alternative explanations of path re-use behaviour, such as site familiarity and spatial knowledge. Lizards established the same number of paths, and used them as often, whether they had occupied their home ranges for one or for more years. We discuss our findings in relation to maintenance of the monogamous mating system of this species, and the spatial and social structuring of the population.

## Introduction

Movement and space use are fundamental aspects of an animal’s life and critical for many ecological processes (Kays et al., 2015; Nathan et al., 2008). Movement determines the level of exposure to environmental conditions, such as heat when moving in or out of shade (Dawson et al., 2006; Firth & Belan, 1998), access to resources, and also interaction frequencies with conspecifics, predators or prey (Arias-Del Razo et al., 2012; Smaldino & Schank, 2012). Similarly, natal dispersal, the movement away from the birthplace to the site of first reproduction, influences exposure to new environments and access to mating partners (del Mar Delgado et al., 2009). Movement paths can either be consistent, with individuals repeatedly moving along similar paths, such as long distance migration routes (Åkesson et al., 2012), or they can be variable, such as when animals like desert ants (*Cataglyphis sp.*) use different, often the most direct, routes when returning from different locations back to some central refuge (Wehner, 2003). Investigating how animals move through their environment will contribute to a better understanding of the key role movement plays for ecological and population processes.

Moving along the same paths is a specialised movement strategy allowing very particular insight into space and home range use. Particular functions of path re-use generate predictions of movement behaviour, and allow deductions about the movement strategy. There are several reasons why animals may establish consistent trails. In complex habitats, regularly used trails may represent paths that require the least energy to move through the environment, or the safest passageway to move between habitat fragments (LaPoint et al., 2013). For example, water filled drainage ditches are consistently re-used paths for the green frog (*Rana clamitans melanota*) as they provide high quality habitat pathways to facilitate movement across mostly unsuitable habitat (Mazerolle, 2005). Corridors and other landscape characteristics may also funnel animal movement consistently along particular pathways. Similarly, for flight paths, topography that creates uplift along canyons and mountain ridges in the Appalachian mountains concentrates movement of the golden eagle (*Aquila chrysaetos*) along consistently re-used routes (Dennhardt et al., 2015). Animals may also learn movement paths to reach, most efficiently and safely, resources that are out of sight at the origin of the path, and then repeatedly follow the same trail (Laland & Williams, 1997). Hence, familiarity with the environment would influence such path re-use behaviour. For instance, wild baboons (*Papio ursinus*) move along highly repetitive routes every day to feed on regularly visited fig trees (Noser & Byrne, 2010).

An alternative explanation for repeatedly moving along the same trails is that it can be part of territory or home range marking behaviour. For example, males of the forest thrush (*Catharus fuscescens*) repeatedly move to and sing at the same locations for up to seven days, and Ethiopian wolves (*Canis simensis*) repeatedly move along the border of their territory and scent mark at an average of three scent sites per kilometre (Sillero-Zubiri & Macdonald, 1998). Another example of path re-use as scent marking behaviour is from the pygmy bluetongue lizard (*Tiliqua adelaidensis*). In this species females, but not males, repeatedly move along the same few paths radiating from their single entrance burrows, and males appear to be lured to the female burrow along those scent trails (Ebrahimi et al., 2015).

In this paper we investigate patterns of path re-use in another lizard to explore if those patterns are best explained by scent marking behaviour, or alternatively by familiarity with the most efficient pathways through the environment, for example to commonly used resources. First, focussing on scent marking, we combine our existing understanding of scent marking behaviour in this species with detailed observations of movement patterns to deduce if scent marking behaviour could explain path re-use movement patterns. Investigating scent marking behaviour in natural conditions and identifying scent mark locations across the landscape for entire populations is challenging, but improves our understanding of factors influencing space use and its consequences for population processes.

If repeatedly moving along the same paths is indicative of scent marking behaviour, there are specific predictions about variation among individuals in movement patterns, and about the spatial location of frequently used paths. Scent marks often inform about the quality of the sender (Carazo, Font & Desfilis, 2007; Martín & López, 2015). In a sexual context those scent marks can be used to discourage mating competitors (intra-sexual context) and to attract mates (inter-sexual context) (Blaustein, 1981; Heymann, 2006). Hence, the *mate attraction and mating competition hypothesis* (Heymann, 2006) suggests that the sex experiencing greater mate choice or stronger intra-sexual competition will scent mark more strongly. If path re-use is for scent marking then that sex should re-use paths more frequently. Regarding the effective placement of scent marks, the *hypothesis of economic scent marking* (Gosling & Roberts, 2001a; Roberts & Gosling, 2001) was developed to put scent marking into a spatial context. It suggests that the location where scent marks are most effective depends on home range size and individual mobility. It predicts that species that occupy large home ranges relative to their mobility will concentrate scent markings in their inner core home range areas (hinterland marking), while those with higher mobility and smaller home ranges will mark their home range boundaries including areas that overlap with neighbouring conspecifics (Gosling & Roberts, 2001a; Roberts & Gosling, 2001).

Many squamate reptiles (lizards and snakes) produce scents, either from their skin or specialised glands, and leave continuous scent trails on the substrate when moving through the environment (Mason & Parker, 2010). They have a well-developed vomeronasal system and, through tongue flicking, readily detect and respond to those olfactory signals (Martín & López, 2015). Those chemical systems play an important role in squamate intraspecific communication, and allow differentiation between sexes (Fenner & Bull, 2011), between familiar and unfamiliar individuals (Aragón, López & Martín, 2003; Bull, Griffin & Johnston, 1999; Bull et al., 2000), between related and less related individuals (Bull et al., 2001) and among females of different sexual attractiveness (Parker & Mason, 2012; Uhrig et al., 2012). Furthermore, chemical signals can play a role in the spatial distribution of a species, and have been shown to influence refuge choice (Scott et al., 2013) and to attract females to male home ranges (Martín & López, 2012). Conversely chemical signals can also function as a repellent. In the Iberian wall lizard (*Podarcis hispanica*), olfactory signals allow individuals to assess and avoid the potential threats from neighbours (Carazo, Font & Desfilis, 2008). And males of the Iberian rock lizard (*Iberolacerta cyreni*) delay and reduce the intensity of agonistic interactions with other males that are perceived, from olfactory signals, as the home range owner (López & Martín, 2011). These examples clearly show the influence of olfactory communication on squamate behaviour.

Our study species, the sleepy lizard (*Tiliqua rugosa* Gray, 1825), is a large herbivorous skink with a wide distributional range in mesic and semi-arid regions of southern Australia (Bull, 1995). It is estimated to live up to 50 years (Bull, 1995) and forms long-term monogamous pair bonds (Bull, 1988; Leu et al., 2015), with partner home ranges overlapping almost completely (Kerr & Bull, 2006a). Lizards do not mate each year, and the strength of the pair bond varies among partnerships and years (Bull, 1994; Bull, 2000). Leu et al. (2010a), Leu et al. (2015) and Leu, Kappeler & Bull (2011) defined lizards as paired if they were recorded close together on more than 10% of observation records, and they, and Godfrey et al. (2012) confirmed that each year there were some adult lizards that remained unpaired.

As in other squamates, sleepy lizards detect and respond to conspecific olfactory cues, which play a prominent role in influencing their behaviour. For instance, previous studies have shown that female sleepy lizards use chemical cues for mother-offspring recognition (Main & Bull, 1996) and to discriminate between their pair partner and unfamiliar males (Bull & Lindle, 2002). Individuals also follow scent trails to locate partners (Bull, Bedford & Schulz, 1993a; Bull & Lindle, 2002). Olfactory signalling by neighbours is a possible mechanism to maintain the reported stable spatial and social organisation with little shift in home range location between years (Bull & Freake, 1999; Godfrey, Sih & Bull, 2013; Leu et al., 2016).

In this study we investigated path re-use behaviour in the sleepy lizard and determined whether it conforms with predictions of scent marking behaviour, or with predictions of site familiarity. The scent marking aspect of our study builds on our previous experimental work which identified that sleepy lizards leave scent trails as they brush against the surface while moving through the landscape and directly respond to them (Bull, Bedford & Schulz, 1993a; Bull & Lindle, 2002). Repeatedly using the same paths would concentrate natural scent trails in certain areas as well as repeatedly refresh those scent marks, thereby reinforcing their signalling strength. From the *mate attraction and mating competition hypothesis* we predicted a higher frequency of path re-use in males than females, because sleepy lizard males experience stronger intra-sexual competition (Bull & Pamula, 1996; How & Bull, 2002; Murray & Bull, 2004). Following from the same hypothesis, we predicted greater competition for mates, and hence greater path following behaviour, in paired compared to unpaired individuals. Although pair bonds are stable over time, not all females reproduce each year (Bull, 1988; Leu et al., 2015), and some adult individuals in one year (more likely to be females) may be unpaired if they are not seeking matings. Then, from the *hypothesis of economic scent marking* (Roberts & Gosling, 2001) we predicted that more re-used paths would be located within the inner than the outer areas of the home range, because sleepy lizard daily movement capabilities are low relative to their home range size (Bull & Freake, 1999; Kerr & Bull, 2006a).

In addition to investigating whether scent marking behaviour can explain path re-use, we also investigated the alternative hypothesis that frequent path re-use results from familiarity with the most efficient routes to areas with commonly used resources, such as patches of high food abundance or important refuge sites. Individuals need time to acquire information of their surroundings, and spatial knowledge increases with settlement duration (del Mar Delgado et al., 2009). In return, familiarity with an area, i.e. spatial knowledge influences individual movement behaviour (Noser & Byrne, 2010). For example, butterfly fishes move along predictable paths when foraging (Reese, 1989) and chipmunks (*Tamias striatus*) run along particular paths to escape predators (Clarke et al., 1993). Stamps (1995) suggested that in species occupying complex habitats, individuals learn and use certain paths to move rapidly and efficiently around obstacles and barriers in familiar areas. Although our study site, in flat, open chenopod scrubland has few obvious environmental constraints on movement that might impede or channel movement along particular trajectories, bushes and interspersed trees may constitute local obstacles that lizards need to move around. We hypothesised that familiarity with the home range area and resource distribution influences movement behaviour in the sleepy lizard and predicted that path re-use behaviour would be more frequent among long-term resident lizards than among lizards that had recently arrived and were less familiar with the area.

## Material and Methods

### Study site

Our study site was an approximate 1.5 × 1.5 km area located near Bundey Bore Station in the mid-north region of South Australia (33°54′16″S, 139°20′43″E), with an average annual rainfall of about 250 mm. It contains relatively homogeneous chenopod scrubland, dominated by blue bush, *Maireana sedifolia*, with interspersed small stands of sheoak trees, *Casuarina cristata*. An infrequently used vehicle track crosses the site but does not impede movement. The habitat structure of the study site is very open, allowing many movement paths, and lizards regularly move across the vehicle track at multiple locations along its length. The study was conducted in the austral spring and early summer (Aug–Dec) of two years, 2009 and 2010. This is the period of year when lizards are most active. It is normally too cool for lizard movement earlier in the year, and movement is inhibited after December when most of the annual plants that they feed on have dried out and food is unavailable. Our previous records suggest little movement activity outside of these study periods (Kerr & Bull, 2006b).

### Data recording

We used previously described procedures (Godfrey et al., 2012; Leu et al., 2010a; Leu, Kappeler & Bull, 2010b; Wohlfeil et al., 2013) to collect lizard GPS location data. In the early spring of each year (August–September) we caught all 60 resident adult lizards within the study site and attached data loggers, each containing a GPS unit, a radio transmitter and a step counter, to the dorsal surface of their tails using surgical tape. The data loggers recorded synchronous GPS locations for all active lizards every 10 min, over the following 4 months. GPS locations were only recorded if a lizard had been taken at least one step in the past ten minutes. Radio transmitters with unique frequencies allowed us to identify and locate each lizard every 12 days to download data and change batteries. Lizards were measured (snout-to-vent length) and weighed at each data download. Each data logger plus radio transmitter weighed 37 g, 4.5% of the body weight of an average adult lizard, or 6.6% of the lightest lizard in our study. We found no evidence that GPS loggers adversely affected lizard behaviour or condition. Repeated measures of mass showed no unnatural decrease in body condition. We observed neither behavioural lethargy when lizards were relocated and caught, nor signs of skin damage where loggers were attached. We removed the GPS loggers and released all lizards at the end of the study. In the following months after their release, sleepy lizards naturally shed their skin which would rid them of any undetected skin damage.

Individual lizards that were followed for fewer than 30 days were excluded from our analyses, leaving observations of 55 lizards (30 males, 25 females) in 2009 and 60 lizards (30 males, 30 females) in 2010. Forty-three lizards (23 males, 20 females) were observed in both years. The study required permanent marking of individual lizards, in order to allow identification across years. We used a toe-clip numbering code analogous to the technique used by Sinn, While & Wapstra (2008), which has been shown to cause low stress in lizards (Langkilde & Shine, 2006; Perry et al., 2011). The study was conducted with a Permit to Undertake Scientific Research from the South Australian Department of the Environment, Water and Natural Resources (permit number A23436). Lizards were treated using procedures formally approved by the Flinders University Animal Welfare Committee in compliance with the Australian Code of Practice for the Use of Animals for Scientific Purposes (permit number E232).

### Description of path use

We divided the study area into more than 22,000 10 m × 10 m grid cells, and considered a lizard was moving along a path if it was in two different grid cells in consecutive GPS readings (10 min apart). We defined a path to have started on the first occasion when consecutive readings were in different cells, and to have ended when an active lizard was recorded in the same grid cell in two consecutive readings. Leu, Kappeler & Bull (2011) reported that the mean distance travelled by an active sleepy lizard was 12–15 m in 10 min, and in this study most paths progressed by one adjacent grid cell between readings. However, a sleepy lizard can occasionally achieve speeds of up to 1.2 km/h (Kerr, Bull & Cottrell, 2004; Main & Bull, 2000) or 200 m in 10 min. Thus we considered that consecutive locations up to 20 grid cells apart were realistic, although this was uncommon. Where consecutively occupied grid cells were not adjacent, we interpolated a direct route between the two GPS readings and deduced the grid cells that the lizard must have passed through on its path.

We determined all paths of each lizard in each year, and then investigated how frequently an individual lizard moved along the same path on separate occasions within the same year. For this analysis we considered all paths that were five grid cells long (equivalent to about 50 m), and included all 50 m subsets of any longer paths. Thus a six cell path sequence ABCDEF would be represented in the analysis as two paths, ABCDE and BCDEF. Although this may have resulted in some instances where more than one sequence came from the same longer path, it was biologically important to include all segments of a re-used path and not arbitrarily truncate the path to one sequence of five cells. Our choice of five cell paths was informed by the average of about 200 m that sleepy lizards travel per day (Kerr & Bull, 2006b; Main & Bull, 2000), as well as the inherent imprecision of the GPS units (Leu et al., 2010a). We matched paths with identical grid cell sequences, but permitted forward and reverse matching, that is movement along the same path but in opposite directions. Then, for each individual in each year we determined how many different paths were re-used at least three times and the total number of times those paths were used (the sum, over all paths, of the number of times each frequently used path was followed).

### Accounting for home range size and the number of GPS locations

The likelihood that a lizard will move along the same path by chance alone should increase with decreasing home range size. We accounted for this relationship by including individual home range size (in hectares) as a covariate in our subsequent modelling. We calculated home range size for each individual in Ranges 8 (Kenward et al., 2008). We used the 95% Minimum Convex Polygon (MCP) that excluded outlier locations while representing the overall area of activity. Additionally, the number of records of path re-use should increase with the number of recorded locations, and we accounted for this by including the number of GPS locations for each individual as a covariate. The number of GPS locations itself is a function of the number of days the lizard was observed and the individual activity level of the lizard on those days, since GPS locations were only recorded when lizards were active and moving.

### Effects of sex and pairing status on path re-use behaviour

We hypothesised that, if a function of path re-use was to establish scent trails, both sex and pairing status would influence path re-use behaviour. At first capture we determined the sex of each individual by the relatively broader heads of males (Bull & Pamula, 1996), and through gently everting the male hemipenes (Bull, 1988). As previously described (Leu et al., 2015; Leu, Kappeler & Bull, 2011), we determined that a male and female lizard were paired if they were within 2 m of each other, as determined by their synchronous GPS locations, on at least 10% of recorded occasions when both were active (Leu et al., 2010a). The 10% contact threshold to define pairing is arbitrary, but is biologically meaningful, because other interactions are usually brief and infrequent while pairing is a prolonged association (Leu et al., 2010a). Infrequently males become paired with more than one female within a season, with those pair partners also repeatedly and consistently interacting (Leu et al., 2010a). Here, we considered both females in those polygynous partnerships to be paired. Females actively participate in the maintenance of the pair bond (Leu, Kappeler & Bull, 2011), and we considered that both females in these relationships may participate in scent marking.

All analyses were performed in IBM SPSS 20. We constructed repeated measures linear models using the mixed function in SPSS, which allows missing data, so we could include lizards that were not tracked in both years. We used sex, pairing status and year as fixed factors, and home range size and number of GPS locations as covariates, as described above. We did not include lizard as a random factor but took the repeated measure structure of our data into account. We used those models to analyse two dependent variables, the number of paths re-used and the total number of times those paths were re-used. We ln(x + 1) transformed both measures of path re-use to achieve a normal distribution of the error terms of the models. First, we built full factorial repeated measures models with different covariance structures and determined the most suitable model covariance structure as compound symmetry. This likens our model to a repeated measures ANOVA but allows for missing data points. We followed West, Welch & Galecki (2007) and compared models that contained the same variables but differed in their covariance structure using restricted likelihood ratio tests, with p-values calculated using χ^2^ distributions (West, Welch & Galecki, 2007). We did not include the 3-way interaction term, as it is often difficult to interpret, after confirming that it did not play a role.

### Re-used path in relation to home range structure

We investigated the locations of the re-used paths, and whether they were predominantly found within the inner or outer parts of the home range. We calculated the 50% minimum convex polygon (MCP) home range of each lizard in each year as the inner home range area. The 50% MCP contained 50 percent of all active locations of each lizard, and hence the likelihood to form paths was the same in the inner and outer home range parts. For each lizard we determined in Ranges 8 (Kenward et al., 2008) the proportion of grid cells of its repeatedly used paths that were located within the 50% MCP home range. We then multiplied the proportion of grid cells with the total number of repeatedly used paths to achieve a measure comparable to the number of paths we had used earlier. Because each path had five grid cells (and was about 50 m long) parts of some paths could have been in both the inner and the outer home range area. Our calculations proportionally assigned those paths to both inner and outer home range areas. We inferred the number of paths in the outer home range area was the difference between the number of paths in the inner home range area and total number of repeatedly used paths. Allowing paths to be partially within the inner and outer home range areas, resulted in a more conservative estimate of the proportion of repeatedly used paths in either area, than if we had omitted paths that were partially in both areas, or if we had assigned paths to the area where they were mostly located. In our analyses we focussed on the re-used paths of the 43 lizards observed in both years. Because we analysed whether re-used paths were predominantly in the inner or outer parts of the home range, lizards that did not re-use any path in 2009 or 2010 were excluded (5 lizards from the 2009 data, and 2 lizards from the 2010 data). As before, we ln(x + 1) transformed the dependent variable and constructed a repeated measures linear model using the mixed function in SPSS, with year and home range part as fixed factors, and home range size and number of GPS locations as covariates. Again we determined the model covariance structure as compound symmetry, but, different from above, we accounted for the second level of repeated measures (year and home range part) when building the model.

### Effect of residency on path re-use behaviour

Finally, we investigating the alternative hypothesis of home range familiarity for path re-use behaviour and explored in a further analysis whether path re-use behaviour was influenced by residency, which we assumed represented longer term familiarity with the area. For this we only used the records from 2010, and we classified all lizards followed in that year as residents if they had been previously caught in 2009, and as new arrivals if they were caught for the first time in 2010. Again, we ln(x + 1) transformed the dependent variables, and constructed two linear models using the mixed function, analysing the number of paths re-used and the total number of times those paths were re-used, and included sex, pairing status and residency as fixed effects, and home range size and number of GPS locations as covariates.

## Results

The composition of the study population in each year is shown in Table 1. We deduced 808 (2009) and 1252 (2010) paths, five grid cells long, that were used at least three times by an individual lizard. Details of the tracking data and path use parameters derived from the GPS locations each year are shown in Table 2. Fifty of the 55 lizards in 2009, and 56 of the 60 lizards in 2010 often (three or more times) moved along paths which they had used before, while five lizards in 2009, and four in 2010 never used the same path more than twice. Although our analyses considered two separate measures of path re-use, results were completely consistent for both.

**Table 1:** Composition of the study population. Number of individuals in each category.

Year	2009	2010
Male	30	30
Female	25	30
Paired	37	37
Un-paired	18	23
Resident		43
New arrival		17

**Table 2:** Average tracking data and path use parameters per lizard. Measures were derived from the GPS locations each year.

Year	2009	2010
Mean (SE) days observed	81.327 (2.067)	86.333 (2.008)
Mean (SE) GPS locations recorded	1634.491 (51.319)	2062.617 (62.123)
Median (min, max) number of re-used paths used	8 (0, 84)	11 (0, 139)
Median (min, max) total re-use frequency	26 (0, 276)	31 (0, 515)

**Figure 1:**
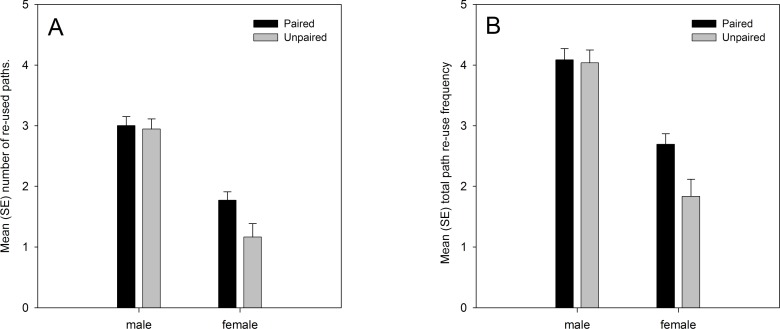
(A) number of re-used paths, and (B) total path re-use frequency in relation to sex and pairing status. Both variables were ln(x + 1) transformed, and means are estimated marginal means from the model.

Analysis of the data from both years showed significant main effects on path re-use behaviour of sex and pairing status. Males re-used their own paths more than females, and paired lizards re-used their own paths more than unpaired lizards (Fig. 1). There was also a marginally significant sex × pairing status interaction effect. Paired females moved along previously used paths more frequently than unpaired females, while the difference between paired and unpaired males was smaller (Fig. 1). The analysis also confirmed the predicted positive relationship with the number of GPS locations (number of re-used paths: regression coefficient b = 0.001, t_101.450_ = 6.182, p < 0.001; total re-use frequency: b = 0.001, t_103.898_ = 6.069, p < 0.001), albeit with low regression coefficient values due to the high numerical values for the number of GPS locations, and the predicted negative relationship with the home range size (number of re-used paths: regression coefficient b = −0.086, t_98.661_ = −2.539, p = 0.013; total re-use frequency: b = −0.090, t_94.859_ = −2.077, p = 0.041) (Table 3). Path re-use behaviour did not differ significantly between years, nor were there any significant interaction effects with year in the analyses (Table 3).

**Table 3:** Repeated measures linear model of the path re-use behaviour. Number of paths used at least three times, total frequency of path re-use (both variables ln(x + 1) transformed).

	Number of paths			Total re-use frequency		
Variable	df	F	*p*	df	F	*p*
Intercept	105.953	3.817	0.053	105.898	6.510	0.012
GPS locations	101.450	38.215	**<0.001**	103.898	36.828	**<0.001**
Homerange size	98.661	6.448	**0.013**	94.859	4.314	**0.041**
Sex	73.763	63.117	**<0.001**	70.976	56.942	**<0.001**
Paired	100.677	4.431	**0.038**	103.020	4.962	**0.028**
Year	71.155	0.051	0.822	70.263	0.116	0.734
Sex × Paired	100.856	3.035	0.085	103.298	3.981	**0.049**
Sex × Year	46.783	0.051	0.823	45.578	0.0002	0.989
Paired × Year	65.957	1.097	0.299	68.006	0.926	0.339

**Figure 2:**
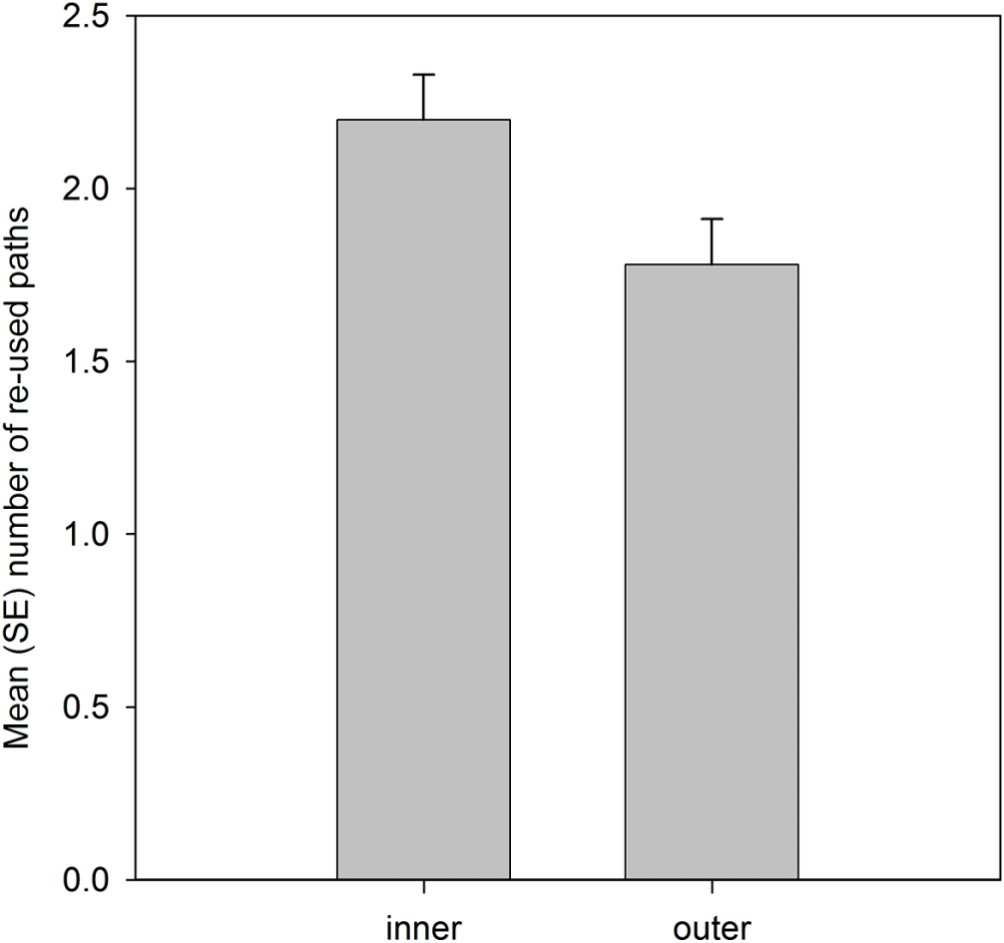
Number of re-used paths, calculated from the five grid cells of each path, that were located in the inner 50% MCP and outer home range area. Variable was ln(x + 1) transformed, and means are estimated marginal means from the model.

Analysis of the spatial locations of the re-used paths showed that significantly more paths were located in the inner 50% than the outer 50% of the home range area (Table 4; Fig. 2).

**Table 4:** Repeated measures linear model of the number of re-used paths in the inner and outer homerange part. Variable was ln(x + 1) transformed.

	Number of paths		
Variable	df	F	*p*
Intercept	150.295	2.603	0.109
GPS locations	138.189	21.641	**<0.001**
Homerange size	122.923	1.879	0.173
Homerange part	70.842	5.096	**0.027**
Year	95.398	0.801	0.373
Homerange part × Year	67.569	0.580	0.449

Analysis that included residency status of the 2010 lizards (Table 5), confirmed the patterns in the previous analysis, but additionally showed there was no significant effect, or any interaction effects, to suggest that familiarity with the site, based on past residency, influenced path re-use behaviour.

**Table 5:** Effect of residency, using 2010 data. Linear model of the path re-use behaviour: number of paths used at least three times, total frequency of path re-use (both variables ln(x + 1) transformed).

	Number of paths			Total re-use frequency		
Variable	df	F	*p*	df	F	*p*
Intercept	51	2.139	0.150	51	4.369	0.042
GPS locations	51	17.475	**<0.001**	51	17.189	**<0.001**
Homerange size	51	1.800	0.186	51	1.179	0.283
Sex	51	50.475	**<0.001**	51	48.229	**<0.001**
Paired	51	9.637	**0.003**	51	11.000	**0.002**
Residency	51	1.582	0.214	51	1.402	0.242
Sex × Paired	51	4.213	**0.045**	51	5.711	**0.021**
Sex × Residency	51	0.326	0.570	51	0.819	0.370
Paired × Residency	51	0.002	0.967	51	0.008	0.931

## Discussion

Our results showed that sleepy lizards commonly established paths within their home range which they repeatedly followed. We also showed there was substantial variation among individual lizards in both the number of re-used paths they established, and in the number of times they used those paths. We deduced that these differences were not related to home range familiarity, because resident lizards did not differ from new arrivals in their patterns of path re-use. Lizard sex and pairing status were more important factors explaining the variation in patterns of path re-use. Males followed their own paths more than did females, and paired lizards followed their own paths more often than did unpaired lizards, particularly among females. Additionally, more re-used paths were in the inner than the outer area of the home range. We explore two explanations for these patterns of variation among individuals in path following behaviour.

First, site familiarity has been shown to affect fitness (Forrester, Casady & Wittmer, 2015). And it was suggested that animals may benefit from site specific knowledge, such as learned locations of important resources and efficient paths that connect these resources (Stamps, 1995; Stamps & Krishnan, 1999). Lizards may establish repeatedly used trails when moving to commonly used resources, such as shelter sites or reliable food patches. In sleepy lizards, shelter sites are often within the inner home range core (Kerr & Bull, 2006a). Similarly, the inner parts of home ranges may include areas with the most commonly exploited food patches. Hence, lizards may have shown greater path re-use behaviour in the inner 50% of the home range where those important resources were located. However, within that interpretation, we expected, but did not find that lizards more familiar with the area would be more likely to know and use common paths. Although this suggests that path re-use does not reflect movement to repeatedly used areas, we cannot entirely reject that hypothesis from our available data. Lizards may acquire spatial information fast enough to become familiar with their central home ranges in their first year, although they most likely arrived in the study area in early spring shortly before we started our observations. While regular use of the same path to reach frequently used resources may be a component of the establishment of repeatedly used paths, we suggest another explanation is more likely.

Our second explanation is that lizards regularly re-use the same paths to establish and maintain a network of scent based signals to indicate their presence to conspecifics. Repeatedly moving along the same path may concentrate those scent trails, and allow them to persist for longer over time. According to this explanation a network of stronger scent marks would be established by path re-use, than by moving more often along different paths within the home range. The presence of strong scent marks would allow lizards to selectively avoid or contact neighbouring individuals (Leu et al., 2010a; Godfrey et al., 2012).

Our observations of variation in path re-use behaviour among individuals are consistent with the predictions of two scent marking hypotheses. First, the prediction derived from the *mate attraction and mating competition hypothesis* (Heymann, 2006) suggests that scent marks are used to attract mates and to repel mating competitors, and that the sex experiencing greater mate choice or stronger intra-sexual competition will scent mark more strongly. In sleepy lizards, intra-sexual conflict is stronger among males than females. Males fight each other by locking jaws (Kerr & Bull, 2002), have wider heads than females, allowing greater bite force in these fights (Bull & Pamula, 1996), and have significantly higher incidence of scale damage, largely around the head and presumably reflecting agonistic encounters (Murray & Bull, 2004). Thus males should scent mark more than females, and this is consistent with our findings of greater path re-use in male than in female sleepy lizards.

If males are the predominant scent markers, it could explain the relatively low level of extra-pair paternity in this species (Bull, Cooper & Baghurst, 1998), despite the lack of persistent mate guarding (Murray & Bull, 2004). Males that are close to their female partner sometimes (but not always) defend her from rival males (Murray & Bull, 2004), but pair partners are only spatially close for an average 30% of their active time during the mating season (Leu, Kappeler & Bull, 2011). Scent marks may supplement physical mate guarding to reduce the opportunity for extra-pair matings.

This still does not completely explain why unpaired males scent marked at a similar, high level to paired males. Sleepy lizards form long-term stable pair bonds (Bull, 1988), and advantages from mate familiarity appear to select for choosing the same pair partner in subsequent years (Leu et al., 2015). The males, which we considered to be unpaired during the study period, could include males from these long-term partnerships with female partners that were not reproducing. A speculative explanation for these findings could be that these males may continue to scent mark to competitors in years when their partner does not reproduce in order to maintain exclusive access to the female partner in subsequent years.

Female scent marking may be to indicate mating readiness to males, as in the related pygmy bluetongue lizard, *Tiliqua adelaidensis* (Ebrahimi et al., 2015). Among females we found that paired females re-use paths more than unpaired females. Females in this long-lived species are unlikely to mate and reproduce every year, because of their high investment into large offspring (Bull, Pamula & Schulze, 1993b; Munns & Daniels, 2007). A proportion of females have only low contact with males in some years, perhaps deferring reproduction until their body condition improves in another year (Bull, 1994; Bull, 2000). This is consistent with our observation that paired females re-use paths more than unpaired females. Females also participate in maintaining partner proximity (Leu, Kappeler & Bull, 2011), and those females that are paired may scent mark more to attract their pair partner and potentially signal mating readiness. Females might also be signalling their mating readiness to neighbouring males. Although extra-pair mating is relatively infrequent in the sleepy lizard (Bull, Cooper & Baghurst, 1998) some females will swap partners, for instance if the male is heavily parasitised (Bull & Burzacott, 2006). These speculations require empirical investigations to determine the intended recipients of female olfactory signalling, but female signalling is likely to be related to mating opportunity, either with the social pair partner or extra-pair males.

An alternative explanation for why paired lizards re-used paths more often could be that unpaired lizards were more exploratory while searching for a mating partner. However, we might predict that some unpaired females are simply not reproducing that year, perhaps due to a lack of reserves following the high energetic investment into offspring in a previous year (Bull, Pamula & Schulze, 1993b; Munns & Daniels, 2007). In that case, unpaired males should show greater increase in exploratory behaviour and a stronger reduction in path re-use than females. Our observed trends were the opposite of that prediction, making that explanation unlikely.

The second scent marking hypothesis, related to *scent marking economics* predicts that scent marks need to be placed around a home range area to maximise detectability by possible intruders, but within economically sustainable constraints (Gosling & Roberts, 2001a; Gosling & Roberts, 2001b). Sleepy lizards have home ranges of about 4 ha (Bull & Freake, 1999) which are large in relation to their daily movement of usually less than 200 m per day (Kerr & Bull, 2006b; Main & Bull, 2000). If one function of path re-use is to scent mark to indicate spatial ownership and to reduce intra-sexual competition for the female partner, then the pattern of higher path re-use within the inner home range area is consistent with the expected strategy of hinterland marking for this species. Hinterland marking is used when the inner home range area is the maximum area that is economically possible to repeatedly mark or when particularly valuable resources are located within this area (Gosling & Roberts, 2001b; Roberts & Gosling, 2001). Although sleepy lizards do not establish exclusive territories, like many other lizard species (Stamps & Krishnan, 1994), they maintain inner core home range areas that are rarely used by other same sex individuals (Kerr & Bull, 2006a). The exclusive inner core area may be partly maintained by scent marks, and path re-use may contribute to that scent marking. Although a previous study found that lizards did not change their overall home ranges when olfaction was experimentally blocked (Zuri & Bull, 2000), that study did not investigate impacts of olfactory blocking on the inner home range structure.

## Conclusions

The results of our analyses are consistent with our hypothesis that path re-use in the sleepy lizard is a form of scent marking behaviour. We have shown that some sleepy lizards repeatedly move along a network of paths, and that the variation in path re-use behaviour between inner and outer sections of the home range, and among different individual lizards is consistent with predictions for scent marking behaviour, and for males to maintain exclusive access to their female pair partners. However, detailed observations of the response of other lizards to the re-used paths, and carefully designed experiments, for example removing or over-marking scent trails on re-used paths could further substantiate our indirect evidence that sleepy lizards re-use paths as scent marking behaviour. Some lizards never used the same path more than twice, while a few others showed low frequencies of path re-use. This may be indicative of a different signalling strategy but remains to be investigated. Furthermore, whether extensive scent marking in males reduces the frequency of extra-pair paternity, and hence has fitness benefits, still needs to be determined. Scent marking is costly, but it could reduce the much higher costs of agonistic interactions to maintain exclusive access to resources including mating partners (Gosling & Roberts, 2001a), and it has the potential to affect the spatial distribution pattern of a population (Martín & López, 2012). If path re-use represents a signalling function in other taxa, then analysis of movement patterns can provide important insights into drivers that can influence the spatial and social structure of a population.

## Supplemental Information

10.7717/peerj.1844/supp-1 Supplemental Information 1 Data 1.

10.7717/peerj.1844/supp-2 Supplemental Information 2 Data 2.

## Additional Information and Declarations

### Field Study Permissions

The following information was supplied relating to field study approvals (i.e., approving body and any reference numbers): The study was conducted with a Permit to Undertake Scientific Research from the South Australian Department of the Environment, Water and Natural Resources (permit number A23436).

### Data Deposition

The following information was supplied regarding data availability: The data can be found in the Supplemental Information.
